# Sea cucumbers of the genus *Stichopus* Brandt, 1835 (Holothuroidea, Stichopodidae) in Straits of Malacca with description of a new species

**DOI:** 10.3897/zookeys.545.6415

**Published:** 2015-12-14

**Authors:** Sau Pinn Woo, Zulfigar Yasin, Shau Hwai Tan, Hiroshi Kajihara, Toshihiko Fujita

**Affiliations:** 1 Department of Natural History Sciences, Graduate School of Science, Hokkaido University, Kita 10 Nishi 8, Sapporo 060-0810, Japan; 2 Institute of Oceanography and Environment, Universiti Malaysia Terengganu, 21030 Kuala Terengganu, Terengganu, Malaysia; 3 Marine Science Laboratory, School of Biological Sciences, Universiti Sains Malaysia, 11800 Minden, Penang, Malaysia; 4 Department of Zoology, National Museum of Nature and Science, Amakubo 4-1-1, Tsukuba 305-0005, Ibaraki, Japan

**Keywords:** Echinodermata, sea cucumbers, *Stichopus*, Malaysia, taxonomy, spicules, shallow reef

## Abstract

Five sea cucumber species including one new species of the genus *Stichopus* are reported from the shallow coral reefs of Straits of Malacca. The new species *Stichopus fusiformiossa* has unusual fusiform spicules in the tentacles, which are not found in the other species of the genus. Pseudo-tables and large perforated plates are newly recorded for *Stichopus hermanni* Semper, 1868 and *Stichopus vastus* Sluiter, 1887, respectively.

## Introduction

Recent revisions on the holothuroid taxonomy have resulted in some taxonomical changes and introduction of several new species in the genus *Stichopus* Brandt, 1835 of the family Stichopodidae (Rowe and Gates 1995; Massin 1999; Massin et al. 2002; Byrne et al. 2010). Outer morphology of *Stichopus* is somehow very deceptive and similar interspecifically (Clark 1922; Massin et al. 2002), and variable intraspecifically. Field identification by their external appearances proved to be difficult and identification using spicules is essential (Clark 1922; Clark and Rowe 1971; Massin et al. 2002; Massin 2007). To enhance the understanding of classification within the family Stichopodidae, Moraes et al. (2004) introduced chemotaxonomic approach while Byrne et al. (2010) and Uthicke et al. (2010) have employed molecular sequence data.

One of the earliest comprehensive records on the diversity and distribution of holothurians in Malaysian waters was done by Ridzwan and Che Bashah (1985). Then, Zulfigar et al. (2008) produced a field guide to sea cucumbers in shallow water and coral reefs in Malaysia. However, taxonomic studies of the genus *Stichopus* in Malaysia were scarce and done only at the southern part of the South China Sea (Siti et al. 1999; Massin et al. 2002). Massin et al. (2002) described two new species, *Stichopus rubermaculosus* and *Stichopus ocellatus* there. The genus *Stichopus* is one of the dominant genera in tropical shallow waters which is an important fishery commodity. Although Straits of Malacca is a major area for stichopodid fisheries, there has not been a comprehensive taxonomic study done on stichopodids in that area. Furthermore, the genus *Stichopus* is taxonomically very confusing due to their similarity between each species in outer appearance and the presence of variations (Massin et al. 2002). In this study, detailed morphological descriptions were done on the species of the genus *Stichopus* including a new species found from the shallow reefs of the Straits of Malacca.

## Material and methods

**Figure 1.**
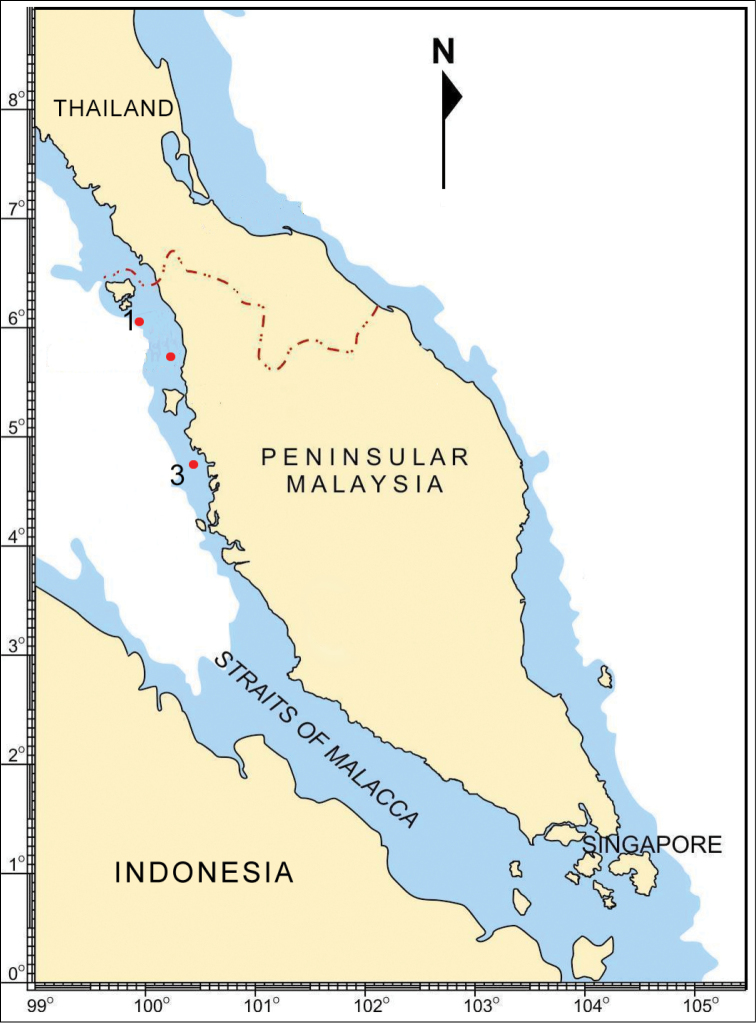
Map of study area in the Straits of Malacca: **1** Pulau Payar **2** Pulau Songsong **3** Pulau Sembilan.

Sea cucumbers were collected from the shallow coral reef areas of Pulau Payar (6°26'2.7"N, 99°40'54.8"E), Pulau Songsong (5°48'31.2"N, 100°17'38.0"E) and Pulau Sembilan (4°1'46.8"N, 100°32'39.7"E) in the Straits of Malacca as shown in Figure 1. The sampling areas were situated in highly sedimented waters of the Straits of Malacca (Chua et al. 2000) with poor reef framework formation (Pillai and Scheer 1974). All sampling areas exhibited similar shallow reef flat at depths about 10-15m with gradual slope of sandy substrate extending to 30m depth. SCUBA diving was employed in collecting specimens using wandering transect covering an area of about 150–200 m^2^,. up to 30 m water depth during day and night. The sea cucumber specimens were fixed in absolute ethanol for two weeks and stored in 70% ethanol. Spicules were extracted from the tissues of the dorsal body, tip of the papillae, tentacles, and tube feet. The tissue were dissolved using commercial bleach and spicules were then washed several times with distilled water before transferring them to a glass slide to be observed under microscope. Pencil drawing of the spicules were done using a drawing tube attached to the microscope. The pencil drawings were then traced on a tracing paper using fine technical pens and digitized by scanning. All specimens were deposited at Marine Science Laboratory, Universiti Sains Malaysia (USM/MSL).

## Results

### Systematics

#### Stichopodidae

Taxon classificationAnimaliaAspidochirotidaStichopodidae

Haeckel, 1896

Stichopus Brandt, 1835

##### Type species.

*Stichopus chloronotus* Brandt, 1835

##### Diagnosis.

Peltate-shaped tentacles at ventral mouth with quadrangular shaped body. Flattened ventral sole with tube feet in ambulacra area. Papillae conspicuous. No cuvierien tubules and no anal teeth or traceable papillae around the cloacal opening. Gonads in two tufts, one at each sides of the dorsal mesentery. Spicules primarily tables, branched rods and C-shaped rods (Clark and Rowe 1971).

##### Species composition.

This genus consists of 14 species. *Stichopus chloronotus* Brandt, 1835; *Stichous ellipes* Clark, 1938; *Stichopus herrmanni* Semper, 1868; *Stichopus horrens* Selenka, 1867; *Stichopus ludwigi* Erwe, 1913; *Stichopus monotuberculatus* (Quoy & Qaimard, 1834); *Stichopus naso* Semper, 1868; *Stichopus noctivagus* Cherbonnier, 1967; *Stichopus ocellatus* Massin, Zulfigar, Hwai & Boss, 2002; *Stichopus pseudohorrens* Cherbonnier, 1967; *Stichopus quadrifasciatus* Massin, 1999; *Stichopus rubermaculosus* Massin, Zulfigar, Hwai & Boss, 2002; *Stichopus fusiformiossa* sp. n. Woo; *Stichopus vastus* Sluiter, 1887

##### Remarks.

The common characteristics of this genus include gonads with two branching tufts (which is a taxonomic character for the family Stichopodidae) and the presence of tables, C-shaped, and S-shaped rod spicules in the tissue (Clark and Rowe 1971). The distribution of the genus *Stichopus* was throughout the tropical and subtropical waters of the Indo-West Pacific region (Clark and Rowe 1971).

#### Stichopus chloronotus

Taxon classificationAnimaliaAspidochirotidaStichopodidae

Brandt, 1835

Figs 2 , 3

Holothuria (Holothuria) quadrangularis Lesson, 1830: 90, pl 31, fig. 1.
Stichopus (Perideris) chloronotus Brandt, 1835: 250.
Stichopus chloronotus ; Selenka 1867: 315, pl. 17, figs 20–24; 18, fig. 25; Pearson 1903: 204; Panning 1944: 30, fig. 3a–e; Loi and Sach 1963: 238, pl. 1, fig. A, pl. VI, fig. 1; Clark and Rowe 1971: 178, pl. 27, fig. 18; Mary Bai 1980: 16, fig. 101; Tan Tiu 1981: 65, pl. 7, figs 1–3; Clark 1984: 99; Féral and Cherbonnier 1986: 94; Cannon and Silver 1986: 27, fig. 4h; Cherbonnier 1988: 146, fig. 60A–O; James and James 1994: 12, pl. VI; Kerr 1994: 163; Rowe and Gates 1995: 323; Massin et al. 2002: 74, figs 1–2, pl. 1A.
Stichopus cylindricus Haacke, 1880: 47.
Stichopus chloronotus var. fuscus Pearson, 1903: 204.
Stichopus hirotai Mitsukuri, 1912: 161.
Holothuria viridis Cherbonnier, 1952: 19–21, fig. 7.

##### Material examined.

Five specimens: USM/MSL/PB004, USM/MSL/PB005, USM/MSL/PB006, USM/MSL/PB007, USM/MSL/PP005.

##### Type locality.

Lugunor Islands, Guam.

##### Description.

**Figure 2.**
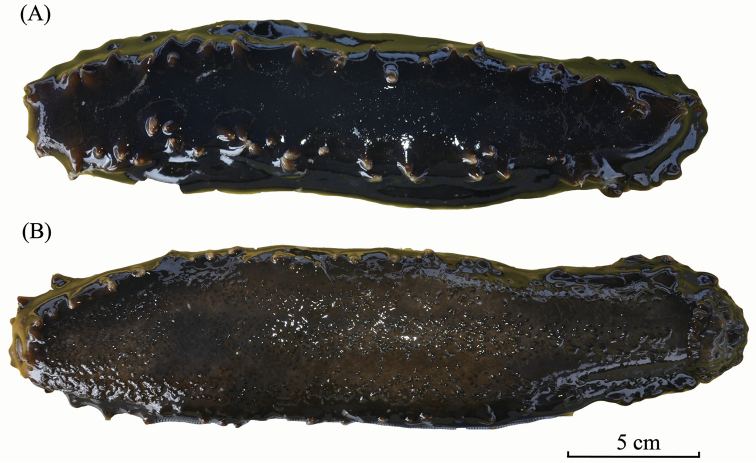
*Stichopus chloronotus* Brandt, 1835 (USM/MSL/PB004), dorsal (**A**) and ventral (**B**) views.

External morphology: Body quadrangular in cross-section with four distinctive sides; smooth, firm, and hard, indicating thick integument; dark blue in colour underwater and almost black out of water (Fig. 2). Large and long papillae at dorso-lateral edge running from collar of tentacles toward anus in two rows; similar papillae at ventro-lateral edge but in one single row; tip to base of papillae yellow to ochre in colour. Ambulacral areas with tube feet and narrow interambulacra; central ambulacrum wider compared to other two ambulacra. Oral opening with 20 peltate tentacles on ventral side; anus at terminal.

**Figure 3.**
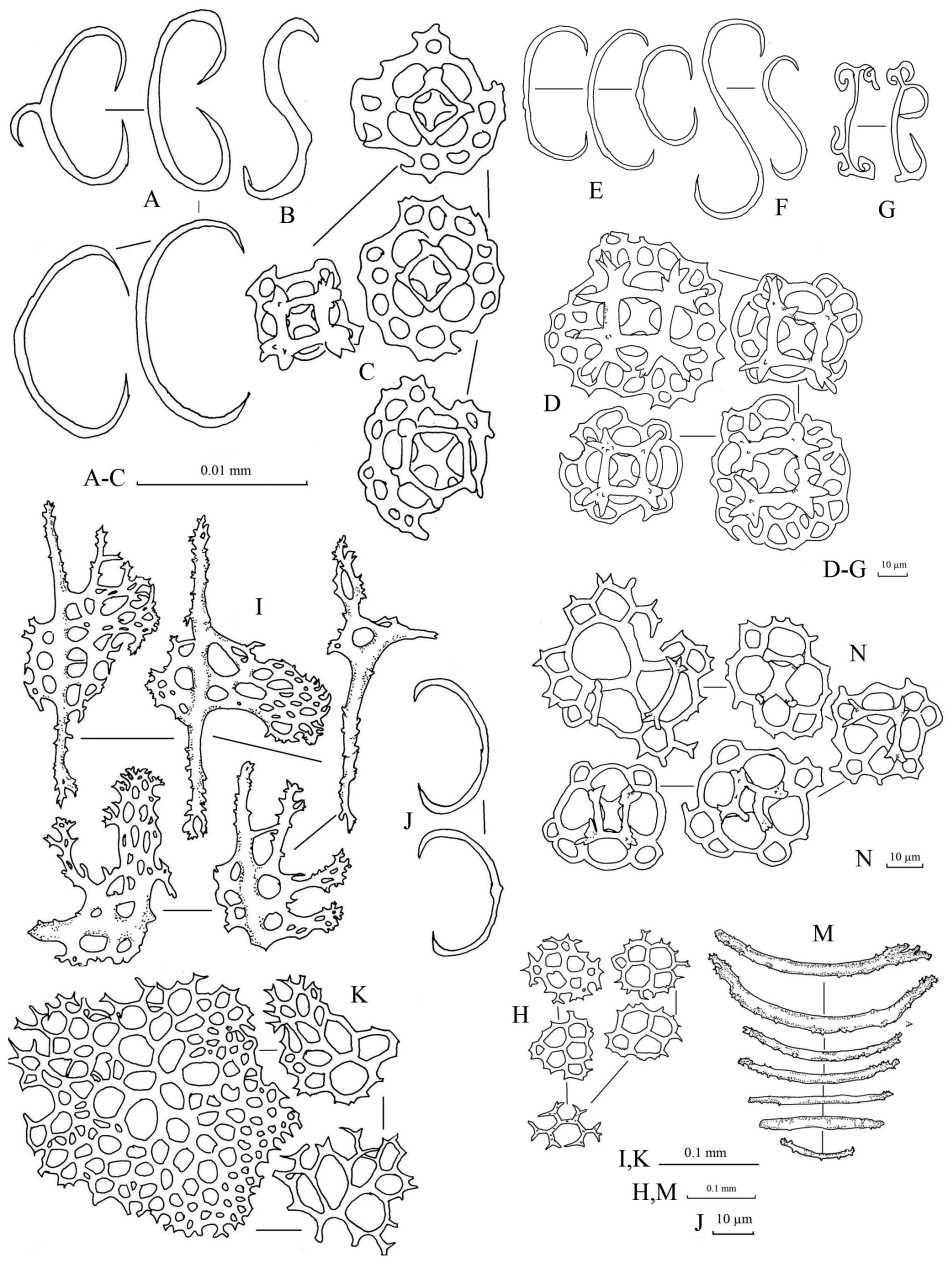
Spicules of *Stichopus chloronotus* Brandt, 1835 (USM/MSL/PB004). **A** C-shaped rods from the dorsal body **B** S-shaped rods from the dorsal body wall **C** tables from the dorsal body wall **D** table with large base from the dorsal papillae **E** C-shaped rods form the dorsal papillae **F** S-shaped rods from the dorsal papillae; G rosettes from the dorsal papillae **H** tables from the tube feet **I** large rods with central perforations from the tube feet **J** C-shaped rods from the tube feet **K** perforated plates from the tube feet **L** reduced tables from the tube feet **M** rods from the tentacles **N** tables from the tentacles.

Spicules: Dorsal body wall with tables, C-shaped rods, and S-shaped rods (Fig. 3A–C). Tables abundant in dorsal body wall; base smooth with four large central perforations and 4–10 smaller peripheral holes; four pillars forming spires, joined with one crossbeam; tip of each pillar spiny. C-shaped rods smooth with sharp endings; some being irregular in form. S-shaped rods derived from C-shaped rods present. Dorsal papillae bearing C-shaped rods, S-shaped rods, elongated rods, and tables (Fig. 3D–G). Tables in dorsal papillae with large disc, four central perforations, and multiple peripheral holes; pillars joined by one, sometimes incomplete, crossbeam; tip of pillars with multiple large spines. Ventral tube feet bearing plates, thick rods, tables, and C-shaped rods (Fig. 3H–J). Large plates in ventral tube feet having distinctive larger central perforations; numerous smaller peripheral holes distributed over plates. Other smaller plates in ventral tube feet having perforations similar in size to each other. Edges of plates in ventral tube feet jagged but less pointy and sharp. Tables in ventral tube feet having reduced pillars or no pillar; one crossbeam incompletely joining all pillars; four central perforations with less peripheral holes compared to tables from dorsal body. Rods in ventral tube feet with 0–3 (mostly one) perforation(s) at central extension (Fig. 3I). Tentacles with tables and rods. Tables in tentacles reduced to only disk without pillars, with four central perforations surrounded by smaller peripheral holes; similar to tables in dorsal body wall (Fig. 3F). Rods in tentacles in various sizes, with spiny and rough surface; smaller rods straight while larger rods slightly curved (Fig. 3L).

##### Remarks.

*Stichopus chloronotus* is fairly easy to be identified in situ due to the bright, distinctive green-blue colouration on the body with yellowish coloured papillae (Fig. 2A), although there are some colour variations in the papillae across different localities (Massin et al. 2002). This species is distinguished from the other congeneric species also by its smooth body.

Large spicules with elaborated sculpted surface in the tentacles reported from Madagascar by Cherbonnier (1988) were not found in our specimens. This type of spicule is also missing from other descriptions by Théel (1886), Massin (1996) and Massin et al. (2002).

##### Distribution.

This is a common species found throughout the Indo-West Pacific area (Clark and Rowe 1971).

#### Stichopus herrmanni

Taxon classificationAnimaliaAspidochirotidaStichopodidae

Semper, 1868

Figs 4 , 5

Stichopus variegatus Semper, 1868: 73.
Stichopus variegatus ; Cherbonnier 1947: 187–189, fig. a–c; Féral and Cherbonnier 1986: 98.
Stichopus herrmanni ; Massin 1999: 63, fig. 52.

##### Material examined.

Four specimens: USM/MSL/PSEM 001, USM/MSL/PSEM002, USM/MSL/PSEM003, USM/MSL/PP004.

##### Type locality.

Philippines.

##### Description.

External morphology: Large body with quadrangular cross-section with four distinctive sides; firm, rugose, and having thick folding surfaces. Uniformly greyish brown on dorsal side; light brown to yellowish on ventral body with an orange patch spreading from mid ventral body to anterior ventral body. Two rows of small and short papillae on dorso-lateral edges; papillae absent on ventro-lateral edges; tip of papillae brown; base grey-coloured. Numerous, smaller, brown-tipped papillae spreading across dorsal body. Laterally, papillae being lesser in number and density. Ring of minute papillae surrounding oral opening. Tube feet numerous in ambulacra areas. Central ambulacrum occupying more rows of tube feet compared to other two ambulacra areas. Narrow interambulacra areas without tube feet. Twenty peltate-shaped tentacles. Anus terminal.

Spicules: Dorsal body mainly tables, C-shaped rods, rosettes, and pseudo tables (Fig. 5A–D). Table spicules in dorsal body have rounded to quadrangular in shape bases, with four central perforations and numerous peripheral holes; three or four short pillars forming spires connected by a cross beam; tip of pillars with thorny crown endings (Fig. 5A). Rosettes in dorsal body abundant with various shapes and sizes; simple to complex bifurcation on both ends (Fig. 5C). Pseudo tables in dorsal body have four pillars extending from reduced base; no disk formed at base (Fig. 5D). Papillae consist of tables, C-shaped rods, and rosettes (Fig. 5E–G). Base of tables in papillae with rough rims; four pillars with multiple spines on the tip erected from disc; four central holes on the disc with 2–3 peripheral holes. C-shaped rods and rosettes in papillae similar to those in dorsal body. Tube feet have large perforated plates, rods, and reduced tables (Fig. 5H–J). Perforated plates in tube feet in rectangular and square shapes, jagged and pointy rims (Fig. 5H). Rods in tube feet with central extended perforations (Fig. 5J); surface covered with spinelets. Reduced tables of tube feet have base with four central perforations and 5–8 peripheral holes; reduced pillars liken knobs formed at central of base; rim of base being smooth (Fig. 5I). Tentacles containing rods of different sizes with rough surfaces covered with spinelets; slightly bended (Fig. 5K).

**Figure 4.**
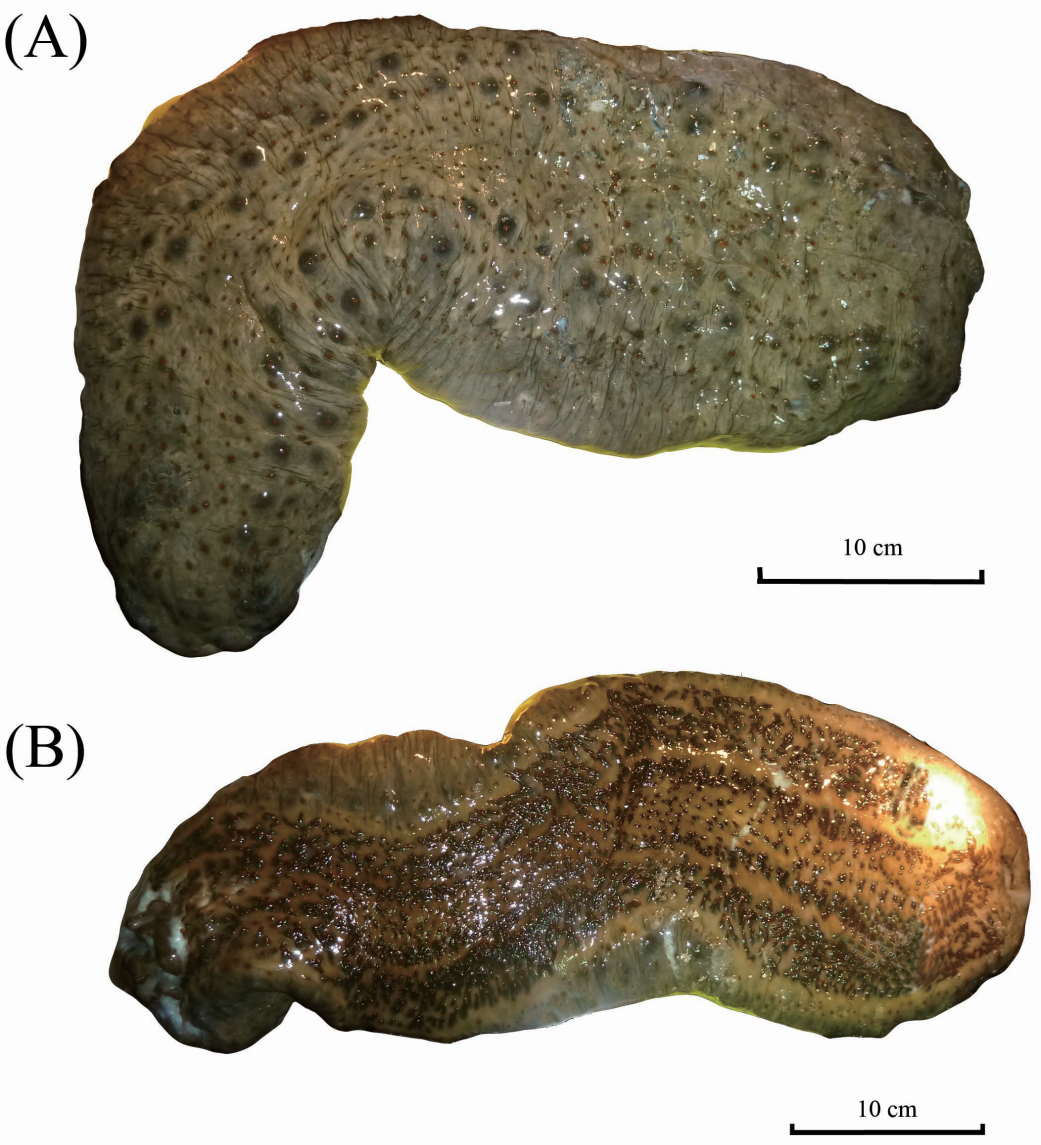
*Stichopus herrmanni* Semper, 1868 (USM/MSL/PSEM004), dorsal (**A**) and ventral (**B**) views.

**Figure 5.**
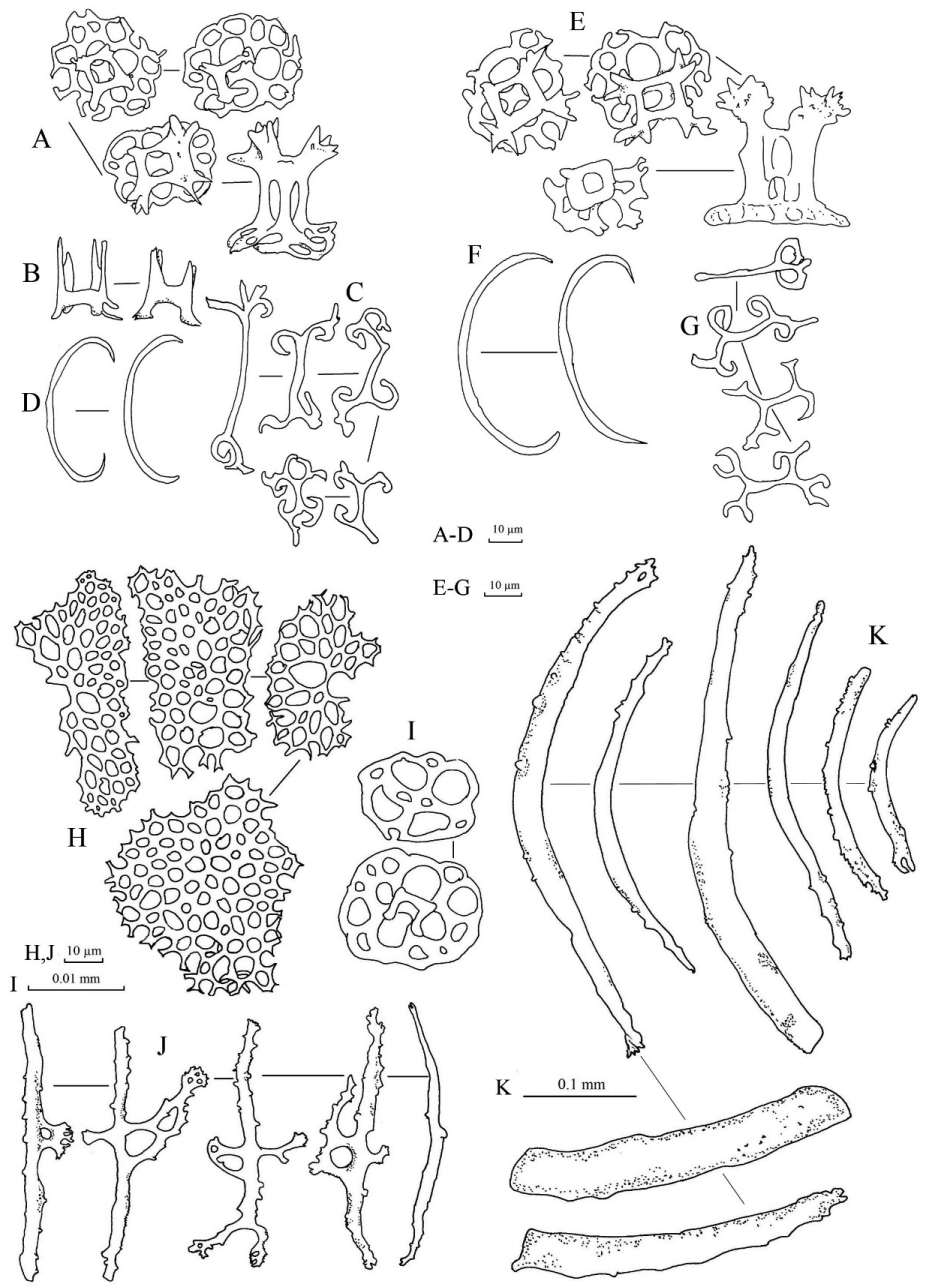
Spicules of *Stichopus herrmanni* Semper, 1868 (USM/MSL/PSEM004). **A** tables from the dorsal body **B** pseudo tables from the dorsal body wall **C** rosettes from the dorsal body wall **D** C-shaped rods from the dorsal body wall **E** tables from the dorsal papillae **F** C-shaped rods from the dorsal papillae **G** rosettes from the dorsal papillae **H** large multiperforated plates from the tube feet **I** reduced tables from the tube feet **J** rods with central perforations from the tube feet **K** rods of different sizes from the tentacles.

##### Remarks.

*Stichopus herrmanni* is originally described as a subspecies of *Stichopus variegatus* before Rowe and Gates (1995) reclassify and accorded *Stichopus herrmanni* a species status. *Stichopus herrmanni* is closely related to *Stichopus monotuberculatus* (Quoy & Gaimard, 1833) but the papillae of *Stichopus herrmanni* are clearly smaller and less conspicuous than those of *Stichopus monotuberculatus*. Massin et al. (2002) noted that rosette spicules in *Stichopus herrmanni* were highly variable in size and abundance. The presence of pseudo-tables found in the dorsal body of our *Stichopus herrmanni* specimens was the first to be reported for the genus *Stichopus*. Pseudo-tables has only been found in *Thelenota* within the family Stichopodidae (Cherbonnier and Féral 1984; Cherbonnier 1988; Massin and Lane 1991; Massin 1999). It is not certain whether this spicule is commonly occurring in this species of different localities. Pseudo-tables can be used to distinguish *Stichopus herrmanni* from other congeners if they are consistently present in specimens from other localities.

##### Distribution.

Throughout the Indo-West Pacific (Clark and Rowe 1971).

#### Stichopus horrens

Taxon classificationAnimaliaAspidochirotidaStichopodidae

Selenka, 1867

Figs 6 , 7

Stichopus horrens Selenka, 1867: 316; Panning 1944: 35; Loi and Sach 1963: 238, pl. 1, fig. B, C, pl. VI, Fig. 2; Cannon and Silver 1986: 27, figs 2d, 7g; Féral and Cherbonnier 1986: 96; Cherbonnier 1988: 147, fig. 61A–P; Rowe and Gates 1995: 324; Gosliner et al. 1996: 281, fig. 1033; Byrne et al. 2010: 1077, fig. 2A–D, fig. 3B–D.
Stichopus godeffroyi Semper, 1868: 75, pl. 30, fig. 4; Sluiter 1901: 31.
Stichopus godeffroyi var. pygmaeus Semper, 1868: 75; Lampert 1885: 105; Ludwig 1888: 812.
Stichopus tropicalis Fisher, 1907: 676, pl. 70, fig. 1a–i.

##### Material examined.

Four specimens: USM/MSL/PP001, USM/MSL/PP002, USM/MSL/PP003, USM/MSL/PP006.

##### Type locality.

Society Islands, French Polynesia

##### Description.

**Figure 6.**
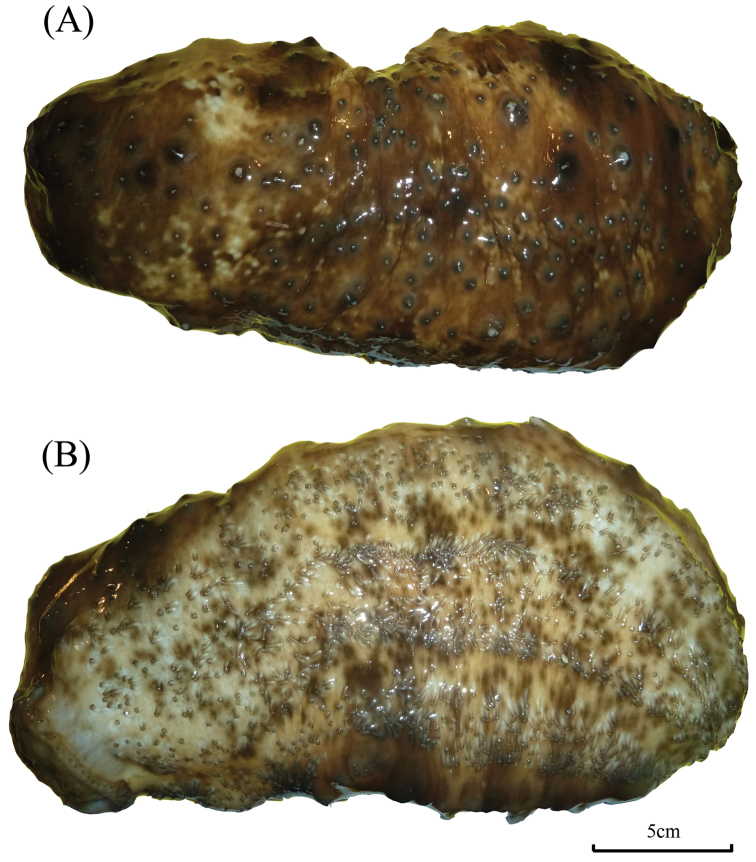
*Stichopus horrens* Selenka, 1867 (USM/MSL/PP001), dorsal (**A**) and ventral (**B**) views.

External morphology: Body slightly sub quadrangular in cross-section, with firm and rough surface; wrinkles on mid-dorsal area. Dominantly dark brown and yellowish in colour, with sporadic whitish-brown patches (Fig. 6). Papillae scattered across dorsal surface with larger papillae along dorso- and ventro-lateral areas; base of papillae dark grey, apex white. Ring of small papillae surrounding oral opening. Tube feet restricted in three ambulacra areas, with mid ambulacrum being wider with more rows of tube feet but a gap of lesser tube feet density in middle area of mid ambulacrum. Cream-white background colour with patches of brown dots across ventral body. Twenty peltate tentacles. Anus terminal.

**Figure 7.**
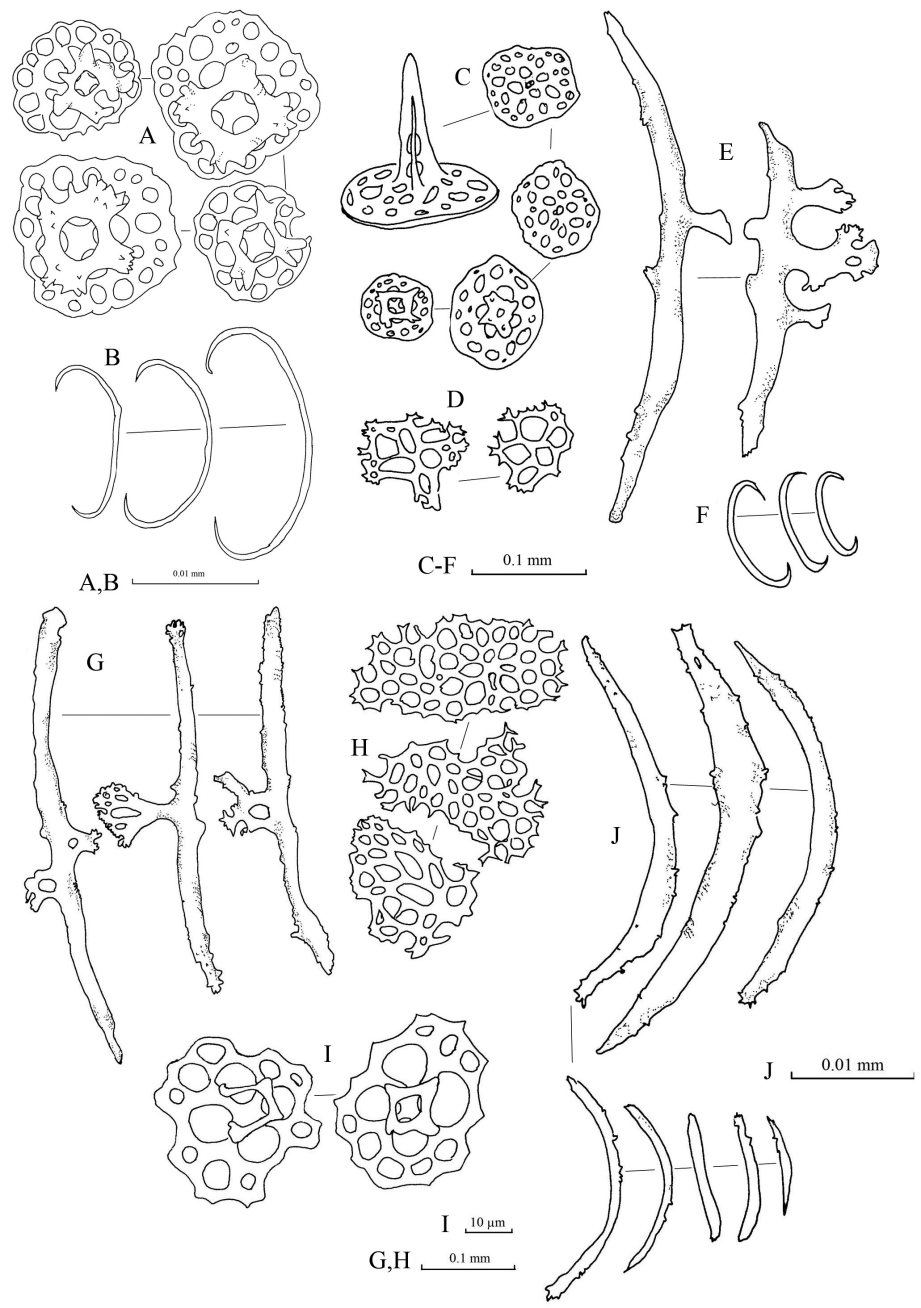
Spicules of *Stichopus horrens* Selenka, 1867 (USM/MSL/PP001). **A** tables from the dorsal body wall **B** C-shaped rods from the dorsal body wall **C** tack-liked tables from the dorsal papillae **D** perforated plates from the dorsal papillae **E** dorsal papillae rods from the dorsal papillae **F** C-shaped rods from the dorsal papillae **G** large rods from the tube feet **H** multiperforated plates from the tube feet **I** tables from the tube feet **J** Rods of different sizes from the tentacles.

Spicules: In dorsal body, numerous tables and C-shaped rods spicules. Tables in dorsal body with four central perforations, 9–21 peripheral holes around smooth-surfaced base; four pillars with moderate heights forming spires joined with one crossbeam, tip of spires with large spines (Figure 7A–B). Spicules in dorsal papillae comprised of tack-liked tables, rods, C-shaped rods, and perforated plates (Fig. 7C–F). Four pillars erected and fused at tips forming tall spire from middle of base (Fig. 7C). Large rods have rough surface and tiny spines on surface, especially at both ends; central perforations can be elaborate or simple (Fig. 7E). Perforated plates in papillae have jagged rims and 6–12 irregular holes; smaller in size than those of tube feet. Tube feet with large rods, multiperforated plates, and tables (Fig. 7G–I). Large rods have central plate with perforations; rod surfaces rough and covered with spinelets (Fig. 7G). In tentacles, spicules consist of rods in different size and thickness (Fig. 7J). All rods have rough surface and spinelets; slight curves in larger rods while smaller appeared straight.

##### Remarks.

This species exhibits diverse body colours in specimens from different localities. Clark (1922) noted specimens from the Hawaiian Islands were dark olive-green, mottled with deep brownish-green in colour, which were not seen in specimens from the Torres Strait. Domantay (1953) remarked that the colour of this species changes with age. The species *Stichopus horrens* look very similar to *Stichopus fusiformiossa* sp. n. but are readily distinguishable by the presence of tack-like spicules on the papillae of *Stichopus horrens* and the absence of fusiform spicules on the tentacles in *Stichopus horrens*. Detailed characters distinguishing these species are provided in the section describing *Stichopus fusiformiossa* sp. n.

##### Distribution.

Society Islands, Galapagos, Indian Ocean, Bay of Bengal, South China Sea, Southern Japan, Papua New Guinea, Philippines, Australia, Hawaii to New Caledonia.

#### Stichopus vastus

Taxon classificationAnimaliaAspidochirotidaStichopodidae

Sluiter, 1887

Figs 8 , 9

Stichopus vastus Sluiter, 1887: 198, pl. 2, figs 46–48; Rowe and Gates 1995: 326; Massin 1999: 71, figs 57a–l, 58a–m, 29a–g, 60a–d, 61, 112d,e; James 1998: 13, fig. 1; Massin et al. 2002: 92, pl. 2E, F, figs 12, 13.
Stichopus spec; Colin and Arneson 1995: 262, fig. 1242; Gosliner et al. 1996: 282, fig. 1039.
Stichopus “variegatus” ; Colin and Arneson 1995: 262, fig. 1240.

##### Material examined.

One specimen, USM/MSL/PLAN001.

##### Type locality.

Java, Indonesia.

##### Description.

**Figure 8.**
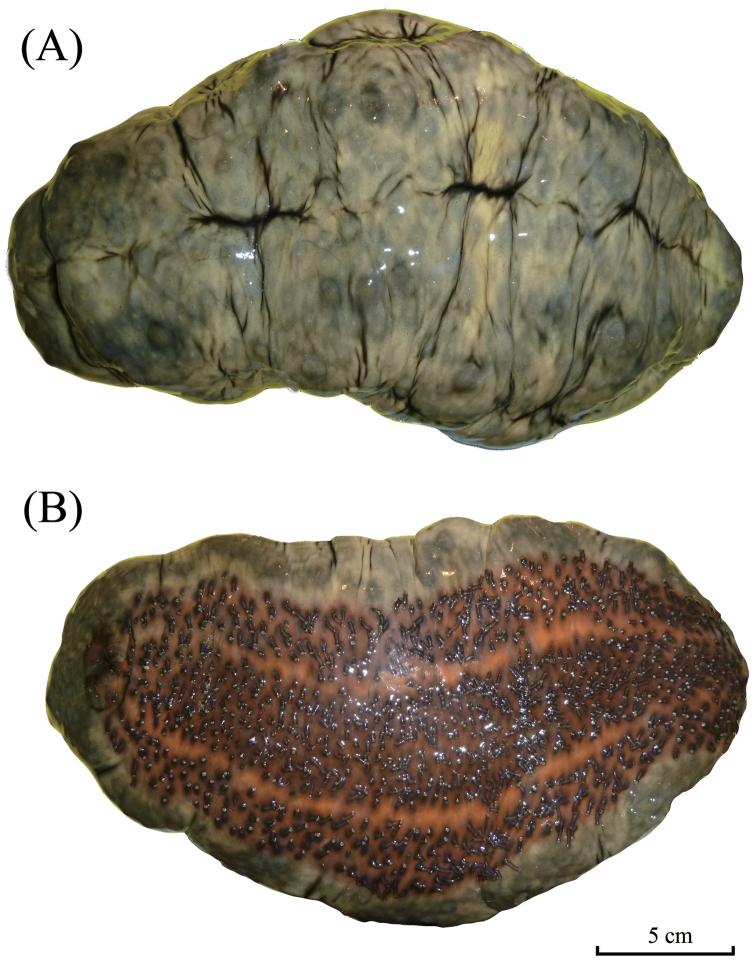
*Stichopus vastus* Sluiter, 1887 (USM/MSL/PLAN001), dorsal (**A**) and ventral (**B**) views.

External morphology: Body slightly quadrangular in cross-section, without distinct edges. Body surface smooth and tough. Black deep depression lines on dorsal body; dorsal background brown colour with numerous darker brown strips transverse and encircles the base of papillae. Two rows of large papillae with very low protrusion on dorsal body. Tube feet only in ambulacral areas; median tube feet twice wider than those in other two ambulacral areas; narrow interambulacral areas clearly separates each ambulacrum area. Reddish brown background colour on ventral side with lighter yellowish-brown on interambulacral areas. Ventral mouth with 18–20 peltate tentacles surrounded by ring of minute papillae at oral opening (Fig. 8).

**Figure 9.**
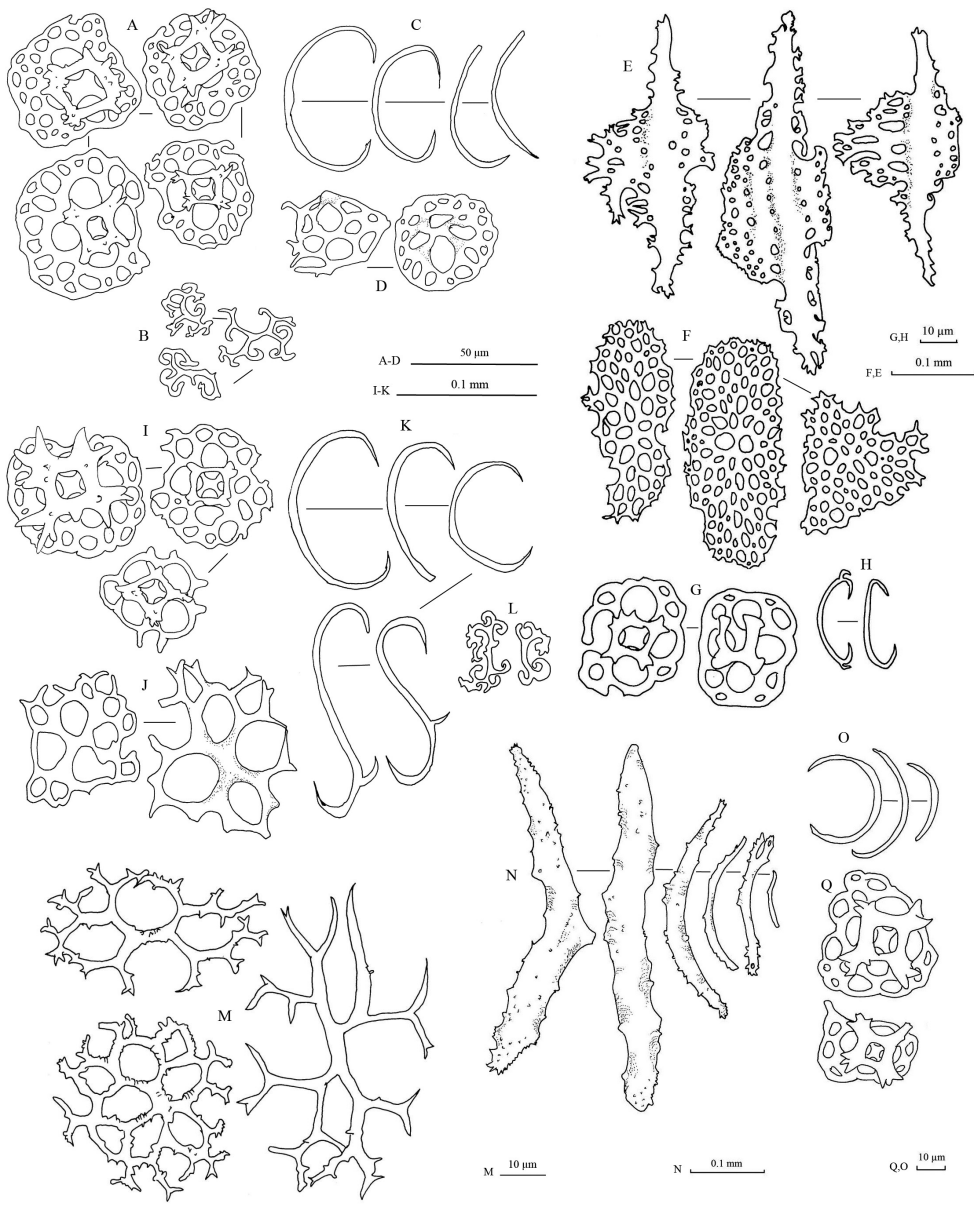
Spicules of *Stichopus vastus* Sluiter, 1887 (USM/MSL/PLAN001). **A** Large tables from the dorsal body wall **B** rosettes from the dorsal body wall **C** C-shaped rods from the dorsal body wall **D** reduced tables from the dorsal body wall **E** rods with large perforated central plate from the tube feet **F** multiperforated plates from the tube feet **G** tables from the tube feet **H** C-shaped rods from the tube feet **I** tables from the dorsal papillae **J** large reduced tables from the dorsal papillae **K** C-shaped and S-shaped rods from the dorsal papillae **L** rosettes found from the dorsal papillae **M** large perforated plates from the dorsal papillae **N** rods from the tentacles **O** C-shaped rods from the tentacles **Q** tables from the tentacles.

Spicule: Tables, C-shaped rod, and rosette spicules present in dorsal body (Fig. 9A–D). Tables in dorsal body with large quadrangular base, four central perforations, and 15–27 smaller peripheral holes; four pillars forming spire from the central of the base with a cross beam connecting them; tip of pillars with multiple thorny spines (Fig. 9A). Reduced tables in dorsal body having similar structure to the base of tables but lack pillars; slightly raised surface on the central (Fig. 9D). Spicule of papillae consists of tables, reduced tables, C-shaped rods, rosettes, and perforated plates (Fig. 9I–M). Tables in papillae similar to those in dorsal body; some with larger thorns on pillar apexes (Fig. 9I). Some C-shaped rods were modified liken S-shaped rods with protrusion at the middle body (Fig. 9K). In ventral tube feet, centrally-perforated rods, perforated plates, tables, and C-shaped rods (Fig. 9E–H) present. Centrally-perforated rods in tube feet have conspicuous large central plate and multiple perforations of different sizes; surface rough and covered with spinelets (Fig. E). Oval- to square-shaped perforated plates; rim were not smooth and jagged (Fig. 9F). Tables in tube feet have small base disc with four main central holes and 5–8 peripheral holes; four pillars erected from central of base with thorny crowns on the tip, but do not extend out of base (Fig. 9G). Small C-shaped rods in tube feet (Fig. 9H). Spicules of tentacles consists of rods, perforated plates, tables, and C-shaped rods (Fig. 9N–Q). Curved rods in tentacles in multiple different sizes; rough surface covered with spinelets especially dense at both ends. Perforated plates in tentacles spiny, rough surface covered with spinelets; some likens dichotomous rods (Fig. 9M). Tables and C-shaped rods in tentacles similar to ones found in the papillae (Fig. 9O, Q).

##### Remarks.

*Stichopus vastus* is the only species in *Stichopus* that have elaborate reticulated stripes covering the dorsal body with different density and intensity (Fig. 8). Massin (1999) and Massin et al. (2002) demonstrated this coloration varies for specimens from across different localities. The black transverse line in the area between bulges is also unique to this species. Massin et al. (2002) mentioned S-shaped rods were found in the dorsal body in small specimens, and the absent in larger specimens (>160 mm in body length). C-shaped rods in the tentacles of the present specimens were also mentioned by Cherbonnier and Féral (1984) but not by Massin (1999) and Massin et al. (2002). Prominent large perforated plates found in the dorsal papillae have not been reported from this species and they look like dichotomous branching rods found in the tentacles.

##### Distribution.

Indian Ocean, Andaman Islands, South China Sea, Indonesia, Thailand, Flores Sea, Great Barrier Reef Australia, Micronesia, Palau, Papua New Guinea (Clark and Rowe 1971; Massin et al. 2002).

#### Stichopus fusiformiossa

Taxon classificationAnimaliaAspidochirotidaStichopodidae

Woo sp. n.

http://zoobank.org/76607F6A-8FF8-4FDA-A5AD-DA0BD359381C

**Figure 10.**
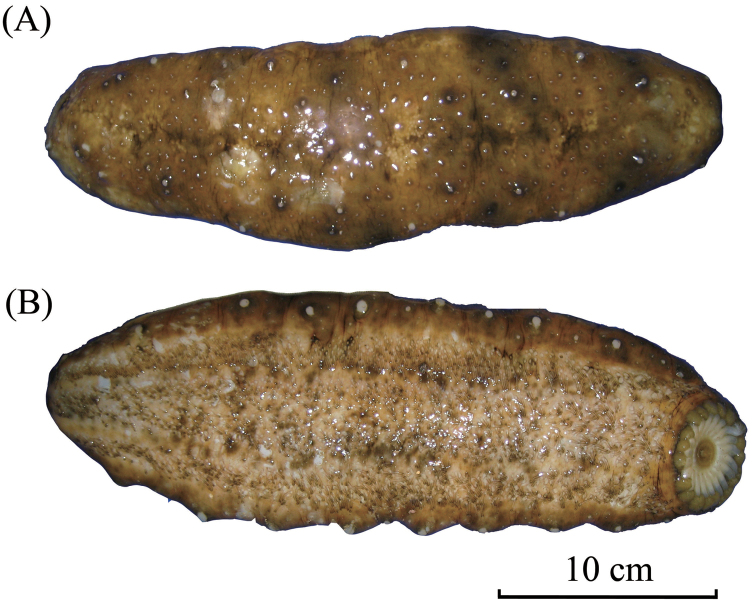
*Stichopus fusiformiossa* sp. n.(USM/MSL/PSS001), dorsal (**A**) and ventral (**B**) views.

**Figure 11.**
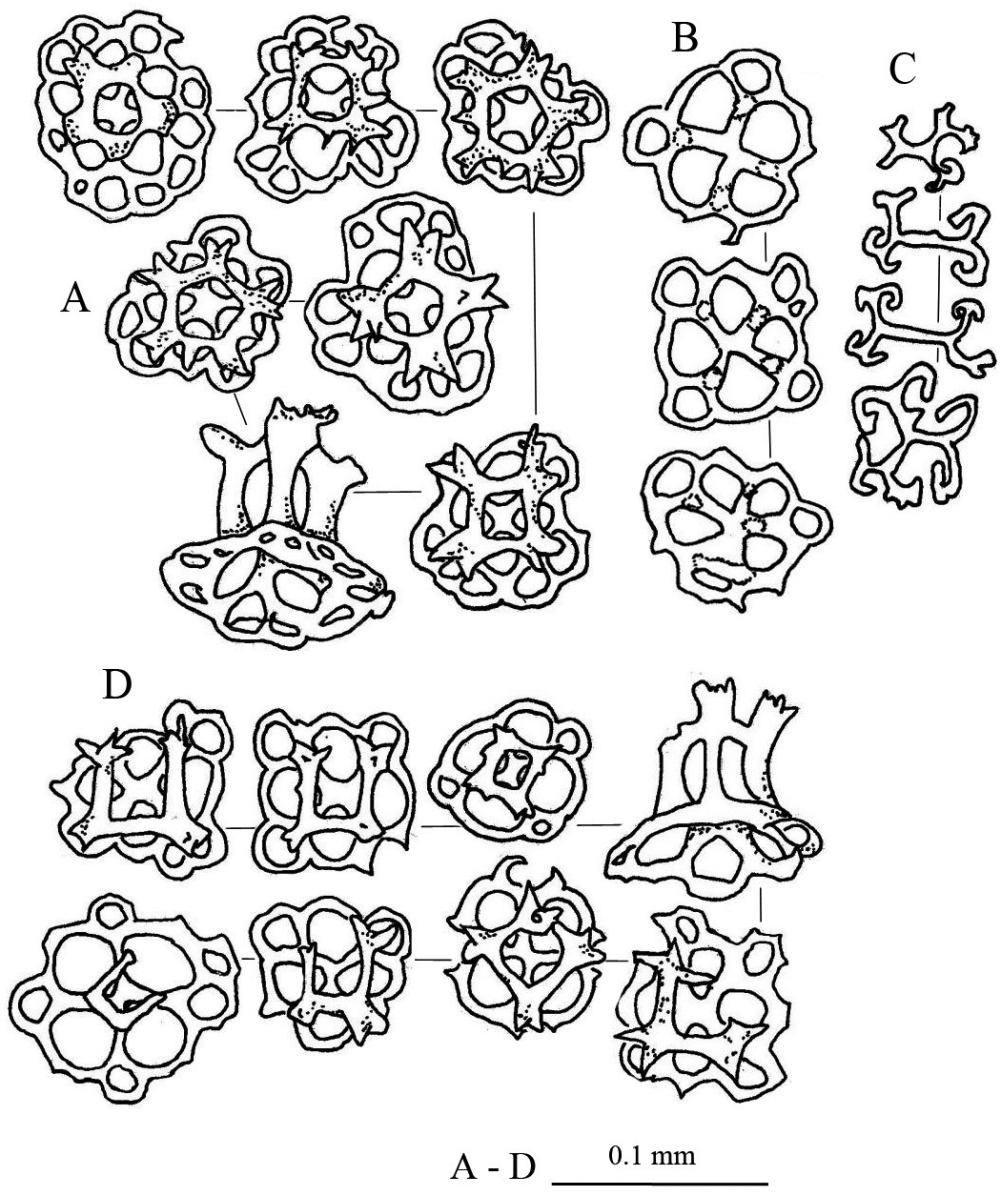
Spicules from the dorsal body wall of *Stichopus fusiformiossa* sp. n. (USM/MSL/PSS001). **A** tables with multiple perforations from the base of the dorsal body wall **B** reduced tables **C** rosettes **D** tables with limited number of perforations from the base of the dorsal body wall.

**Figure 12.**
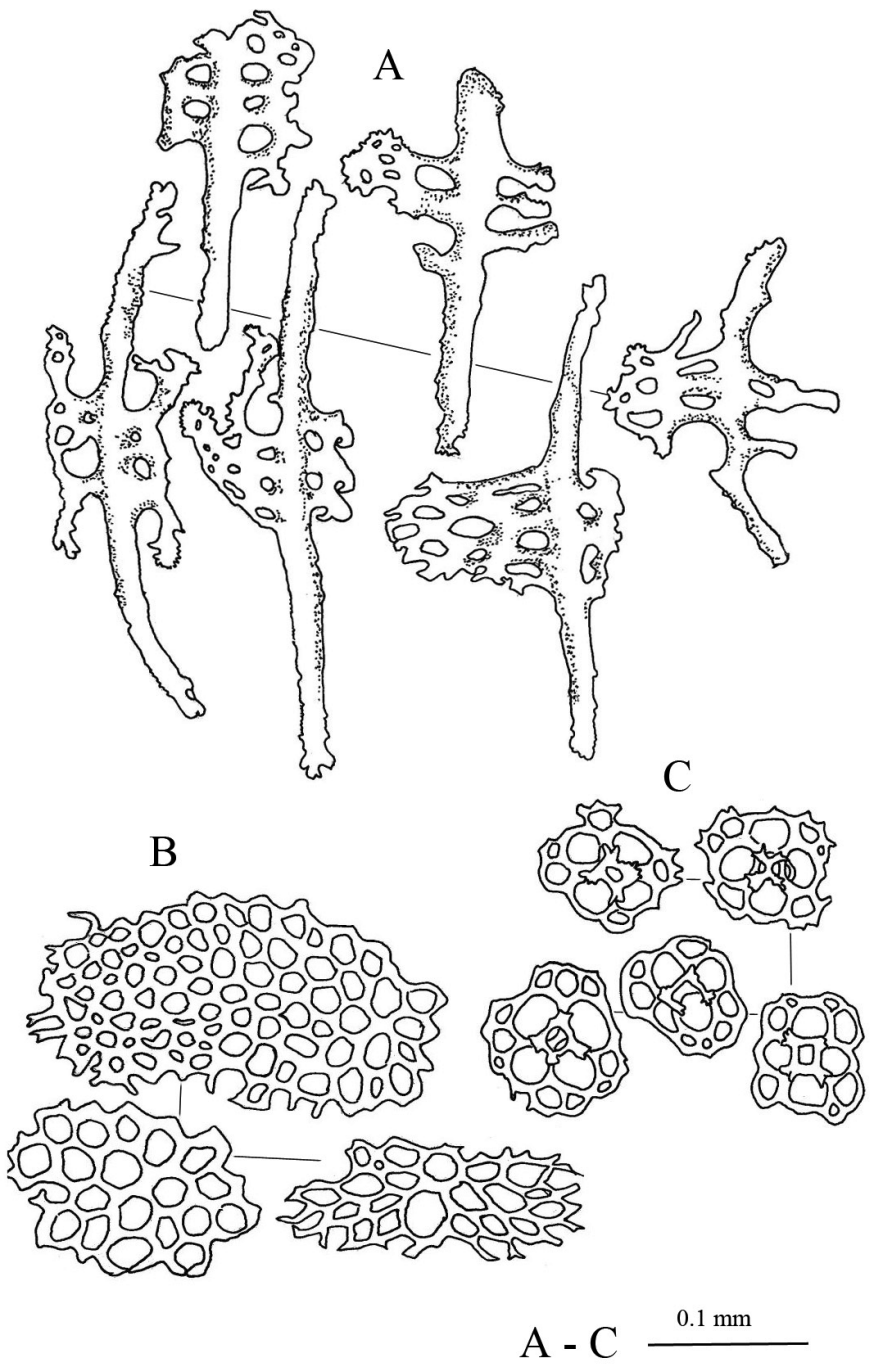
Spicules from the tube feet of *Stichopus fusiformiossa* sp. n. (USM/MSL/PSS001). **A** rods with large central perforated plates **B** large perforated plates **C** tables.

**Figure 13.**
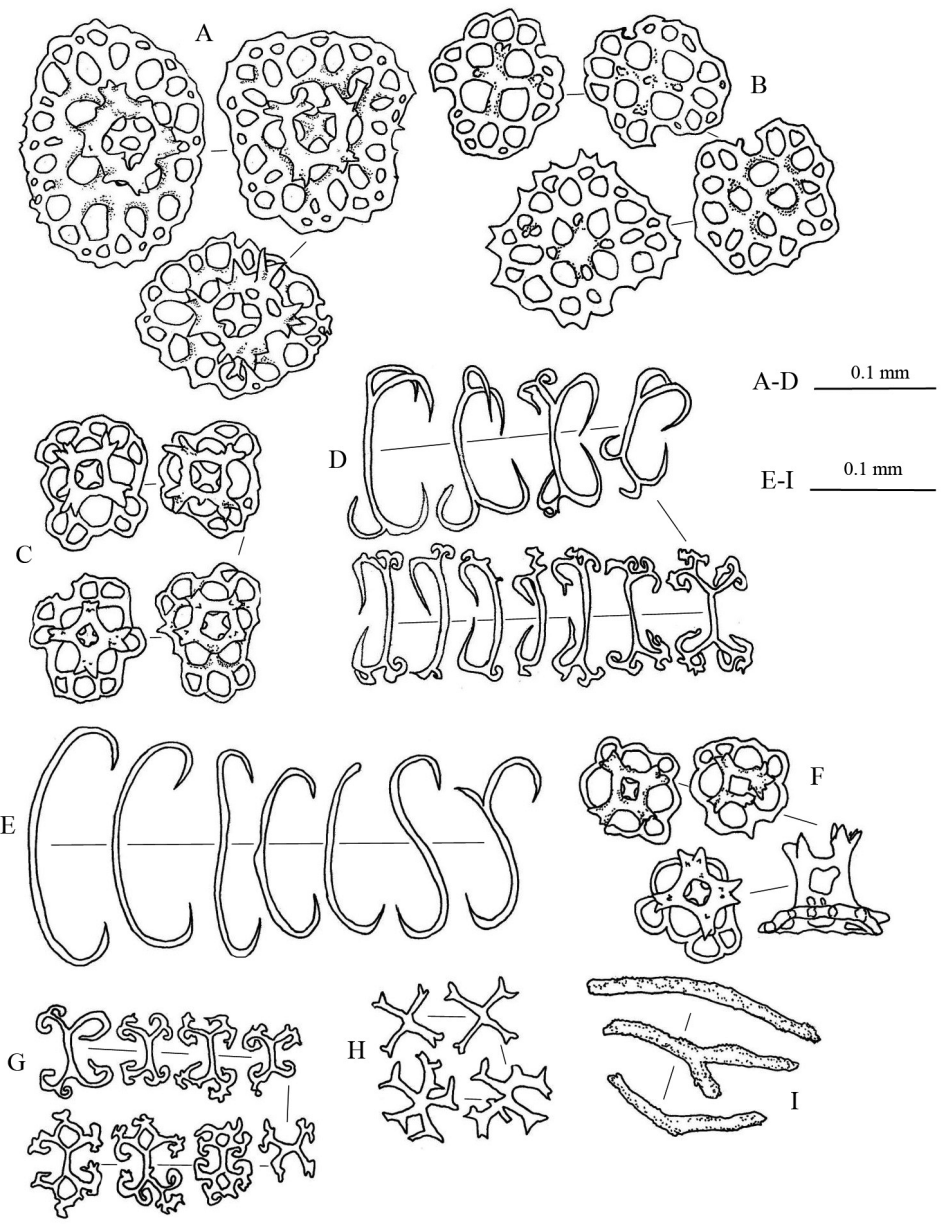
Spicules from papillae of *Stichopus fusiformiossa* sp. n. (USM/MSL/PSS001). **A** table with large base plates **B** reduced tables **C** tables **D** modified C-shaped rods **E** C-shaped rods **F** tables with smaller base plates and number of perforations **G** rosettes **H** X-shaped rods **I** rods.

**Figure 14.**
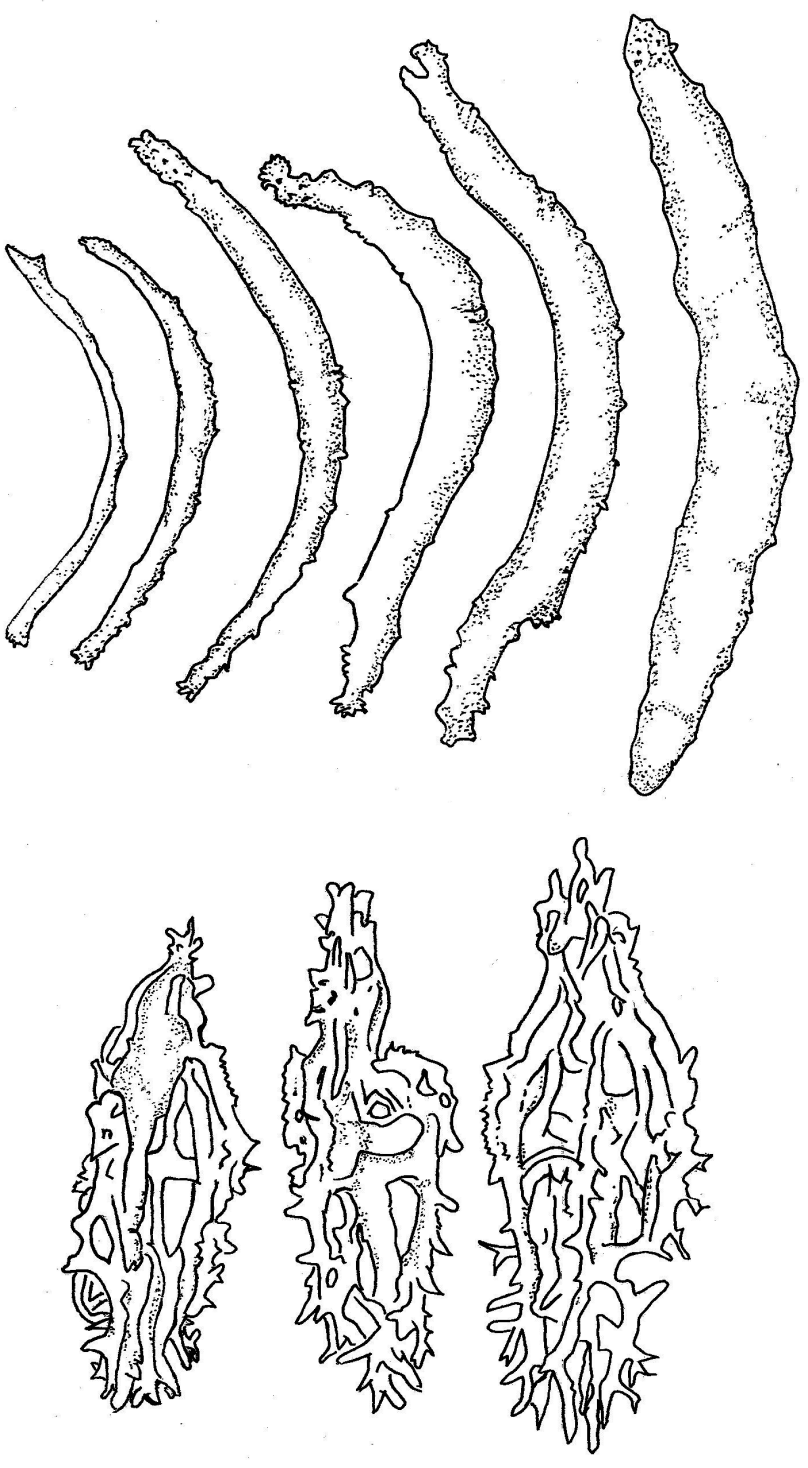
Spicules from the tentacles of *Stichopus fusiformiossa* sp. n. (USM/MSL/PSS001). **A** curved rods **B** fusiform spicules.

Figs 10 , 11 , 12 , 13 , 14

##### Material examined.

Three specimens: Holotype, USM/MSL/PSS001, collected from Pulau Songsong (5°48'31.2"N, 100°17'38.0"E ), Kedah, Malaysia, 6m depth, on sand, fixed in 99% ethanol. Paratype 1, USM/MSL/PSS002, collected from Pulau Songsong (5°48'31.2"N, 100°17'38.0"E ), Kedah, Malaysia, 8m depth, on sand, fixed in 99% ethanol. Paratype 2, USM/MSL/PP003, collected from Pulau Songsong (5°48'31.2"N, 100°17'38.0"E ), Kedah, Malaysia, 7m depth, on sand, fixed in 99% ethanol.

##### Type locality.

Straits of Malacca, Malaysia: Pulau Songsong, Kedah, 05°48'31.2"N, 100°17'38.0"E, on sandy substrate adjacent to a reef area, at a depth of 6–8 m, collected by Woo SP and Zulfigar Y.

##### Description.

External morphology: Body quadrangular in cross-section with slight rounded four sides. Body wall firm, rugose, and wrinkled surface; variously-sized tiny warts regularly-arranged on dorsal body (Fig. 10). Dorsal body brown in background colour, with patchy beige areas and black patches; latter usually running from anterior to posterior ends and concentrated in middle part of body, and absent in some specimens. Ventral body wall light beige background with two orange, narrow lines spreading from oral to aboral. Large dorsal papillae 0.5–1.0 cm in width at base in the holotype, but highly variable between specimens, more or less arranged in two continuous rows; smaller dorsal papillae scattered on dorsal body; colour of dorsal papillae black, with apex always white. Two rows of large (about 2 cm), white-coloured papillae aligned and arranged in straight line along ventro-lateral edges. Minute papillae forming ring surrounding oral opening. Tube feet in ambulacral areas, more numerous in central ambulacral areas than in lateral ones. Two interambulacral areas very narrow, with lesser number of tube feet. Tube feet fairly long, 3–5 mm in length. All specimens examined having 20 peltate-shaped tentacles. Anus terminal.

Spicules: Spicules in dorsal body consisting tables and rosettes (Fig. 11A–D). Many tables in dorsal body having large base with four central perforations and multiple peripheral perforations; four pillars erected from base forming a spire connected by a cross beam, with spines at the tip (Fig. 11A). Some tables in dorsal body having smaller base with less peripheral perforations; pillars connected incompletely with a crossbeam, tip of spire without crown of spines (Fig. 11D). Tables in dorsal body sometimes with pillars reduced to knobs and disc with four central perforations and limited peripheral perforations (Fig. 11B). Papillae consisting large tables, reduced tables, rosettes, C-shape rods, X-shaped rods, and rods (Fig. 13A–I). Large tables in papillae with multiperforated base disc; four pillars forming spire connected with a crossbeam, tip of pillar very spiny (Fig. 13A). Reduced tables in papillae larger compared to reduced tables in dorsal body (Fig. 13B). Rosettes in papillae form simple curving to extensive bifurcations at both ends (Fig. 13D); smaller rosettes more complex in bifurcations (Fig. 13G). C-shaped rods in papillae simple, some modified to S-shaped rods (Fig. 13E). X-shaped rods in papillae have bifurcate endings in each arms; some with five arms (Fig. 13H). Rods in papillae with rough surface (Fig. 13I). Spicules in tube feet mainly large rods with perforated central plate, multiperforated plates, and tables (Fig. 12E–G). Surface of rods in tube feet rough, covered with spinelets; numbers and sizes of perforations on central plates of tube feet vary between rods. Tables in tube feet simpler compared to tables in dorsal body and papillae; tip of pillars less spinous and forming small crowns. Spicules in the tentacles consisting of fusiform spicules and rods (Fig. 11A, B). Fusiform spicules spindle-like in shape with dense spines interconnected to each other forming some hollow spaces in between (Fig. 14B). Rods in tentacles rough, slightly curved, and densely covered with spinelets at both ends.

##### Remarks.

This new species looks very much like *Stichopus horrens* in its body colouration. They both have similar, grey-brown background with irregular grey and black spots in colour. The live specimens of this species do not have very long papillae as observed in *Stichopus horrens* reported by Rowe and Gates (1995) and Massin et al. (2002). *Stichopus fusiformiossa* also lacks tack-like table spicules in the papillae which are the definitive taxonomic feature for *Stichopus horrens*. The prominent white and black colour of the papillae is distinctive to separate *Stichopus fusiformiossa* from *Stichopus rubermaculosus* with the red-coloured papillae, *Stichopus quadrifasciatus* that has brown to red papillae tip, and from *Stichopus chloronotus* with yellow-coloured papillae. Furthermore, *Stichopus quadrifasciatus* has four transverse black-grey bands on the dorsal body wall, which are not seen in *Stichopus fusiformiossa*. The sporadic arrangement of papillae on the dorsal body of *Stichopus fusiformiossa* do not have consistent nor specific patterns as similarly observed in *Stichopus horrens* in this study. However, the two rows of large, black papillae with white tip, arranged in a straight line along ventro-lateral edges are not seen in any other species.

*Stichopus fusiformiossa* lacks C-shaped rods in the dorsal body, which is commonly seen in the other *Stichopus* species. Reduced tables are found in the dorsal body in *Stichopus fusiformiossa* and *Stichopus herrmanni*, but the former lacks rosettes and C-shaped spicules that are present in the dorsal body of *Stichopus herrmanni*. The C-shaped rods in the papillae of *Stichopus fusiformiossa* are strongly modified and do not resemble to any spicules observed in the other *Stichopus* species except the C-shaped rods of *Stichopus chloronotus* (Theel 1886; Sluiter 1887; Ludwig 1887; Mitsukuri 1912). But the colouration and arrangement of papillae easily separate *Stichopus fusiformiossa* from *Stichopus chloronotus*.

The X-shaped rods in the papillae of *Stichopus fusiformiossa* have been only found in *Stichopus variegatus* (now *Stichopus herrmanni*) by Cherbonnier (1988). The X-shaped rods of *Stichopus variegatus* (now *Stichopus herrmanni*) are derived from rosettes (Cherbonnier 1988). Careful examination of his drawings (Cherbonnier 1988: fig. 62H) suggested that X-shaped rods of *Stichopus fusiformiossa* were more rigid and angled compared to the curvy and slender ones of *Stichopus variegatus* (now *Stichopus herrmanni*).

The thick rods with rough surfaces seen in *Stichopus fusiformiossa* are commonly found in the tentacles of any other species of *Stichopus*. Fusiform and spindle-liked spicules instead are rare and only shared with *Stichopus variegatus* (now *Stichopus herrmanni*) (Cherbonnier 1947, fig. C). Since *Stichopus variegatus* had been separated to either *Stichopus herrmanni* and *Stichopus monotuberculatus* by Rowe and Gates (1995), the presence of fusiform and spindle-like spicules can be accorded to *Stichopus fusiformiossa* as a character differentiating it from both *Stichopus herrmanni* and *Stichopus monotuberculatus* because both *Stichopus herrmanni* and *Stichopus monotuberculatus* lack the presence of this spicule. A reexamination of specimens of Cherbonnier (1947) collected from the Gulf of Oman, Madagascar, and the Red Sea are necessary to establish the correct species name of those specimens.

##### Etymology.

The new specific name is a compound descriptive name from the combination of adjective-noun derived from the Latin words of fusiform (fusiformis) and bone (ossa). The name is nominative, neuter, and plural; referring to the distinctive fusiform and spindle-liked spicules found in the tentacles.

### Key to the species of *Stichopus* in Straits of Malacca

**Table:** 

1	Tack-liked tables present in dorsal papillae	***Stichopus horrens***
–	Tack-liked tables absent in dorsal papillae	**2**
2	Fusiform spicules present in tentacles	***Stichopus fusiformiossa***
–	Fusiform spicules absent in tentacles	**3**
3	Uniform blue green colouration on dorsal body wall	***Stichopus chloronotus***
–	Beige, brownish to yellowish colouration on dorsal body.	**4**
4	Elaborate reticulated stripes on the dorsal body wall	***Stichopus vastus***
–	No stripy body wall	***Stichopus herrmanni***

## Supplementary Material

XML Treatment for Stichopodidae

XML Treatment for Stichopus chloronotus

XML Treatment for Stichopus herrmanni

XML Treatment for Stichopus horrens

XML Treatment for Stichopus vastus

XML Treatment for Stichopus fusiformiossa
